# Assessment of TB underreporting by level of reporting system in Lagos, Nigeria

**DOI:** 10.5588/pha.22.0008

**Published:** 2022-09-21

**Authors:** M. Gidado, E. M. H. Mitchell, A. O. Adejumo, J. Levy, O. Emperor, A. Lawson, N. Chukwueme, H. Abdur-Razak, A. Idris, A. Adebowale

**Affiliations:** 1 KNCV Tuberculosis Foundation, The Hague, Netherlands; 2 Department of Public Health, Institute for Tropical Medicine, Antwerp, Belgium; 3 Mainland Hospital, Yaba, Lagos, Nigeria; 4 National TB & Leprosy Control Program, Abuja, Nigeria; 5 New York Medical College, NY, New York; 6 Health Research Unit, Lagos State Ministry of Health, Lagos, Nigeria; 7 Lagos State TB and Leprosy Control Program, Lagos State Ministry of Health, Lagos, Nigeria; 8 Walden University, Minneapolis, MN, USA

**Keywords:** TB underreporting, inventory study, TB reporting system, Nigeria

## Abstract

**BACKGROUND:**

Nigeria has an estimated TB prevalence of 219 per 100,000 population. In 2019, Nigeria diagnosed and notified 27% of the WHO-estimated cases of all forms of TB and contributed 11% of the missing TB cases globally.

**OBJECTIVE:**

To assess TB underreporting by type and level of health facility (HF), and associated factors in Lagos State, Nigeria.

**METHODOLOGY:**

Quantitative secondary data analysis of TB cases was conducted in 2015. χ^2^ test was used to assess the association between treatment initiation, TB underreporting, local government area (LGA) and HF characteristics.

**RESULTS:**

Overall, 2,064 persons with bacteriologically confirmed TB (15.5%) were not matched to patients in sampled TB registers. Treatment status was unknown for 86 cases (IQR 55–97) per LGA. LGAs with higher case-loads had higher proportions of cases with unknown TB status. Discrepant reporting of treated TB was also common (60% HFs). Primary-level TB treatment facilities and unengaged private facilities were less likely to notify.

**CONCLUSION:**

There was TB under-reporting across all types and levels of HFs and LGAs. There is a need to revise or strengthen the process of supervision and data quality assurance system at all levels

## Abstract (fr)

**CONTEXTE:**

Le Nigeria a une prévalence de la TB estimée à 219 pour 100 000 habitants. En 2019, le Nigéria a diagnostiqué et notifié 27% des cas estimés par l’OMS de toutes les formes de TB et a contribué à 11% des cas de TB manquants dans le monde.

**OBJECTIF:**

Évaluer la sous-déclaration de la TB par type et niveau d’établissement de santé (HF), et les facteurs associés dans l’État de Lagos, au Nigeria.

**MÉTHODES:**

Une analyse quantitative des données secondaires des cas de TB a été réalisée en 2015. Le test χ^2^ a été utilisé pour évaluer l’association entre l’initiation du traitement, la sous-déclaration de la TB, la zone de gouvernement local (LGA) et les caractéristiques des HF.

**RÉSULTATS:**

Dans l’ensemble, 2 064 personnes ayant une TB confirmée par épreuve bactériologique (15,5%) n’ont pas été appariées à des patients dans les registres de TB échantillonnés. Le statut de traitement était inconnu pour 86 cas (IQR 55–97) par LGA. Les LGA ayant un plus grand nombre de cas avaient une plus grande proportion de cas dont le statut de traitement était inconnu. La déclaration discrète de la TB traitée était également fréquente (60% des HF). Les établissements de traitement de la TB de premier niveau et les établissements privés non engagés étaient moins susceptibles de faire des déclarations.

**CONCLUSION:**

Il y avait une sous-déclaration de la TB dans tous les types et niveaux d’HF et de LGA. Il est nécessaire de réviser ou de renforcer le processus de supervision et le système d’assurance qualité des données à tous les niveaux.

TB reporting is reporting diagnosed TB cases from all care providers to relevant health authorities and, ultimately, the WHO.^1^ This process involves people, processes, and tools with clear roles and responsibilities, standard definitions, and guidelines.^2^,^3^ TB underreporting refers to TB cases diagnosed and documented in registers of health facilities (HFs), but not captured at the district or state levels.^4^,^5^ Globally, in 2019, there were 2.9 million missing persons with TB.^6^ This was attributed to under-diagnosis, underreporting and challenges with TB estimates.^5^,^6^ Five countries, including Nigeria, account for over 50% of missing TB cases.^6^

TB reporting is part of the WHO standards of TB care (WHO Standard #27),^7^ which states that “all providers must report both new and retreatment TB cases and their treatment outcomes to the national public health authorities based on applicable legal requirements and policies.” TB underreporting is a symptom of a broader public health surveillance problem and functionality of the general health system. TB under-reporting is context-dependent, and the magnitude varies between nations and within the same country, ranging from about 15% in European countries, 20% in Africa, 30% in the Eastern Mediterranean, and 50% in Asia, with a large private sector.^8^

Nigeria is categorized as a high TB, TB-HIV, and multidrug-resistant TB (MDR-TB) burden country with an estimated TB burden of 219 per 100,000 population.^6^,^9^ The country accounts for 4.4% of the global burden of TB and contributes 11% to all missing TB cases.^6^ In Nigeria, there is low TB service coverage, as only 30% of all existing HFs (both public and private) provide TB services, and the private sector constitutes less than 5% of these TB facilities.^9^ According to the WHO, the country’s case detection rate for all forms of TB and MDR-TB in 2019 was 27 and 11%, respectively.^6^

The poor performance of the Nigerian National TB Program (NTP) (with 73% missing TB cases) is attributed to low TB service coverage. This may result in under-diagnosis, underreporting of existing TB service providers, underreporting from the private sector, and ineffective coordination between the TB reporting system and the country’s surveillance system.^9^–^11^ This dire situation prompted the urgent need to estimate TB underreporting in the country for better prevention and control planning.

To tackle this issue, the Lagos State TB and Leprosy ControI Programme, in collaboration with the US Agency for International Development (USAID) and the NTP, conducted an inventory study in Lagos State in 2017 using the 2015 TB data. This study assessed TB underreporting by level of TB reporting system (from HFs to the local government area [LGA] and State TB Programme) in Lagos, Nigeria, using secondary data from the inventory study.^12^

## METHODS

### Study setting

Lagos State is one of Nigeria’s most densely populated states, with 12 million people.^13^ It has a population growth of 600,000/year, a poverty rate of 64.1% (with US$1 per/day), an unemployment rate of 19.5%, and adult literacy for both genders above 85%.^14^ The estimated TB burden of Lagos is 32,850 (based on population prevalence). The state has 20 LGAs (districts or administrative units) further divided into 57 local council development areas (subunits of a LGA).^15^,^16^ Healthcare services are predominantly provided by private HFs, comprising 87.3% of all HFs in the state.^7^

### Coordination of the TB program in Lagos State

Lagos State commenced DOTS services for TB management in 2003 and introduced a public-private mix for TB management in 2008. However, only 23.8% of TB DOTS centers are located in private HFs, and the private sector’s contribution to the 2019 TB case notification was 25%.^9^,^15^–^17^ TB case notification for Lagos increased from 78/100,000 in 2017 to 90/100,000 in 2019, above the national aggregate of 60/100,000 population.^9^ The State TB Coordinator organizes the State TB Programme activities, the LGA TB supervisor manages the LGAs TB program, and each TB treatment facility has a TB focal person.^15^,^18^

**FIGURE 1.**
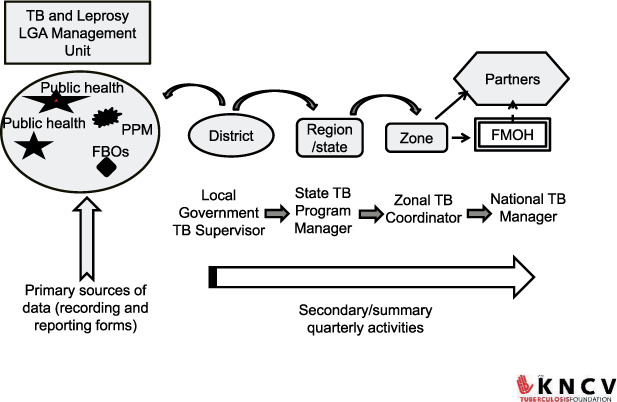
TB reporting system: overview of TB M&E system in Nigeria. LGA = local government area; PPM = private-public mix; FBO = faith-based organization; FMOH = Federal Ministry of Health; M&E = monitoring and evaluation.

Figure 1 depicts the TB reporting process, including levels—HFs, local government (LG) TB registers, the State TB Programme, and the NTP. The TB focal person in HFs is responsible for the day-to-day patient management, including recording of all treatment details of TB patients at the facility in the TB facility register (case-based data). The LG TB supervisor visits all HFs providing TB services within the LGA record and updates case-based data collected by the TB focal person in the LG TB central register, including treatment outcomes.^15^,^18^

The LG TB supervisor uses the quarterly TB case-finding form to report aggregated data to the State TB Programme. The State quarterly report (aggregated) is finally submitted to the central office of the NTP.^15^,^18^ The TB program has structured feedback and a data quality assurance mechanism through supervision, regular data quality visits, and quarterly data review meetings to validate the TB data.^18^ The final reporting of the cohort of patients enrolled for treatment is usually completed a year after enrollment.

### Data extraction

The methodology of the Lagos inventory study and data collection process has been published elsewhere.^12^ For this study, HF variables extracted for analysis from the database of the inventory study were case notification status, the volume of DOTS centers per LGA, engagement with the NTP, type of facility (public vs. private), level of HF (primary vs. secondary vs. tertiary). The laboratory, TB facility register, and LGA registers were digitalized during the inventory study.

### Operational definitions

1. Complete reporting: in this study, a HF was deemed to have complete reporting if there is no difference between the number of TB cases in the TB HF register and the LGA TB register/State TB aggregated data.
2. A facility is underreporting when the number of TB cases in the facility TB register is more than the number of TB cases in the LGA TB register/or State TB aggregated data.
3. A facility is categorized engaged with NTP when the State TB program has a memorandum of understanding with and supports the HF with TB medicines, laboratory supplies, and TB recording and reporting tools. These facilities are documented in the State TB program directory. They are obligated to report all TB cases to the State TB program.
4. A non-engaged HF is a facility not supported by the State TB program.
5. Based on the existing TB facility directory of the state and purposefully using a mean across the LGAs, the volume of TB treatment facilities in a LGA was graded high when the number of TB treatment facilities was ⩾11 (the median), and low when it was below the median number of treatment facilities per LGA.

### Data analysis

The existing database was assessed for the availability of appropriate variables. The level of measurements of all variables was specified by type and level of HFs. TB reporting (dependent variable) was a binary categorical variable categorized as complete reporting and underreporting. All appropriate data sets were extracted to the Statistical Package for Social Sciences (SPSS) software v22 (IBM Corp, Armonk, NY, USA) for analysis. The data obtained were summarized using descriptive statistics such as percentages, mean and median based on the level of measurement of the variables. χ^2^ test was used to assess the association between the dependent categorical variables (TB complete reporting and TB underreporting), and all categorical independent variables.^19^ To determine the TB treatment status of individuals with bacteriologically confirmed TB, all laboratory registers in the state were compared with all engaged DOTs facilities and a sample of unengaged facilities. Facility case-based registers were compared with LG case-based registers to determine the completeness of HF reporting. TB patients in the facility register not in the LGA register were classified as underreported, while individuals found only in the laboratory register were classified as treatment status unknown. For all statistical tests, *P* < 0.05 was considered statistically significant, and 95% confidence intervals were generated for all variables.

### Ethics approval

The Federal Ministry of Health (FMOH) and NTP approved the secondary analysis of the Lagos inventory study data; the primary study was approved by FMOH/Walden University Institutional Review Board (IRB). The Walden University Institutional Review Board (Minneapolis, MN, USA) also approved the study design and analysis.

## RESULTS

**FIGURE 2.**
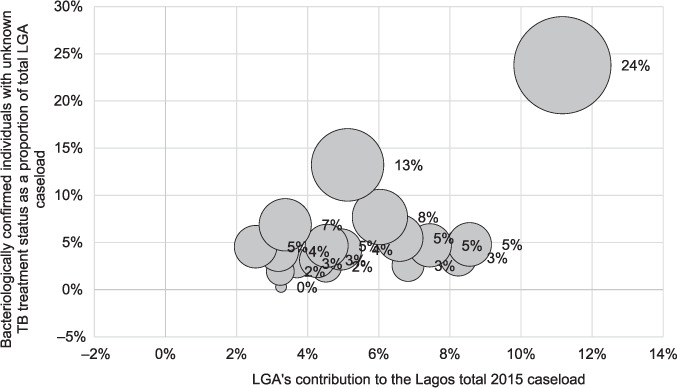
Relationship among successful TB treatment linkage, LGA caseload, and contribution to state notification. LGA = local government area.

There were 2,064 persons with bacteriologically confirmed TB whose TB treatment could not be ascertained, representing 15.5% of all TB cases in 2015. LGAs with larger TB caseloads had larger proportions of patients not treated for TB in engaged facilities or sampled unengaged facilities (Figure 2).

A total of 304 (87%) HFs had documented TB cases in the TB facility registers, of which 258 (84.9%) had reported TB cases in the LGA TB register. Table 1 shows that about 60% of the HFs were public, of which 82.5%, 14.8%, and 2.7% were primary, secondary, and tertiary healthcare facilities, respectively. A total of 9,350 TB patients were treated in sampled facilities in 2015. Private HFs contributed 12.4% of all the cases (Table 2). Less than 40% of all HFs had complete TB reporting to the LG TB registers, with a mean percentage difference of 7.4% (*n* = 649).

**TABLE 1:** Frequency distribution of types and level of healthcare facilities

Variables	*n*	%
Type of facility		
Public	183	60.2
Private	121	39.8
Total	304	
Level of public health facilities		
Primary	151	82.5
Secondary	27	14.8
Tertiary	5	2.7
Total	183	
NTP engagement status		
Engaged	261	85.9
Non-engaged	43	14.1
Total	304	

NTP = National TB Programme.

**TABLE 2:** Number of diagnosed TB cases and number notified in 2015

	Bacteriologically confirmed, treatment status unknown (*n* = 2,067)		Diagnosed and treated (*n* = 9,350)		Diagnosed, treated, notified (*n* = 8,701)	
			
	*n*	%	*n*	%	*n*	%
NTP engagement status of referring facility						
Engaged facilities	1,851	89.0	9,190	98.3		
Unengaged facilities	213	10.3	160	1.7		
Referring or treating facility type						
Public facilities	1,088	82.1	8,188	87.6		
Private facilities	187	14.1	1,162	12.4		
Public health care facility level (*n* = 8,188)						
Primary level	367	27.7	4,285	45.8		
Secondary level	744	56.1	2,975	31.8		
Tertiary level	168	12.7	928	9.9		
Sex						
Male	1,276	61.7	5,492	58.7		
Female	760	36.8	3,858	41.3		
Age group, years						
<15	57	2.8	582	6.2	522	6.3
15–24	342	16.5	1,578	16.9	1,413	17.0
25–34	676	32.7	2,619	28	2,348	28.2
35–44	395	19.1	2,216	23.7	1,987	23.9
45–54	239	11.6	1,248	13.3	1,118	13.4
⩾55	358	17.3	1,067	11.4	932	11.2

NTP = National TB Programme.

Table 3 gives the differences in the aggregated TB data between the HF records as the primary data source and LGA and State TB data. It also shows the LG TB data and State program aggregate data per LG area. The percentage difference in aggregate data between the LG TB registers and the State TB program was less than between HF TB registers and the LG TB registers, with an average of 1% overreporting. Although the difference between LGA TB and State program data was slight, it reflected a mix of underreporting (8 LGAs), overreporting (8 LGAs) and complete reporting (4 LGA).

**TABLE 3:** TB reporting by LGA

LGA ID	Bacteriologically confirmed, status unknown		Proportion of all diagnoses	Total patients facility register		Total patients notified to LGA		Proportion notified to LGA	State notifications to national	Difference between LG TB register and state TB aggregate data
				
	*n*	%	%	*n*	%	*n*	%	%	*n*	%
1	51	2.5	10.8	422	4.5	407	4.7	0.96	398	2.2
3	99	4.8	11.0	800	8.6	710	8.2	0.89	710	0
4	70	3.4	8.3	770	8.2	705	8.1	0.92	713	−1.1
5	84	4.1	22.0	297	3.2	268	3.1	0.90	268	0
6	94	4.6	28.4	237	2.5	219	2.5	0.92	212	3.2
7	53	2.6	7.7	638	6.8	644	7.4	1.01	642	0.3
8	0	0.0	0.0	164	1.8	154	1.8	0.94	152	1.3
9	43	2.8	11.1	346	3.7	310	3.6	0.90	319	−2.9
10	0	0	0.0	72	0.8	71	0.8	0.99	71	0
11	142	6.9	31.1	315	3.4	287	3.3	0.91	287	0
13	273	13.2	36.3	479	5.1	417	4.8	0.87	412	1.2
14	113	5.5	15.5	616	6.6	550	6.3	0.89	576	−4.7
15	88	4.3	16.0	462	4.9	409	4.7	0.89	416	−1.7
16	97	4.7	12.2	695	7.4	682	7.8	0.98	680	0.3
17	491	23.8	32.0	1,044	11.2	1,025	11.8	0.98	1059	−3.3
18	96	4.7	18.5	424	4.5	407	4.7	0.96	417	−2.5
20	159	7.7	22.0	564	6.0	546	6.3	0.97	541	0.9
21	65	3.1	14.0	399	4.3	362	4.2	0.91	358	1.1
22	6	0.3	1.9	304	3.3	261	3.0	0.86	262	−0.4
23	40	1.9	11.7	302	3.2	267	3.1	0.88	277	−3.7
Median	86	4	13	423	5	407	5	0.91	405	0.00
Total	2,064			9,350	100.00	8,701	100.00		8,770	−0.8

LGA = local government area.

Among the cohort of people with TB who began TB treatment, there was no association between TB underreporting and type or level of HF and the volume of DOTS centers per LGAs. However, there was an association between NTP engaged status and under-reporting (Table 4).

**TABLE 4:** Associated factors with TB underreporting by reporting levels

Variables	Complete reporting frequency (%)	Underreporting frequency (%)	*P* value
Type of HCF (*n* = 248)			
Public	56 (36.6)	97 (63.4)	0.327
Private	29 (30.5)	66 (69.5)	
Level of public HCF (*n* = 153)			
Primary	49 (36.8)	84 (63.2)	0.549
Secondary	7 (38.9)	11 (61.1)	
Tertiary	0 (0.0)	2 (100.0)	
NTP engagement status (*n* = 248)			
Engaged	85 (39.7)	129 (60.3)	<0.001
Unengaged	0 (0.0)	34 (100.0)	
Volume of DOTS centers per LGA (*n* = 214)			
Low	17 (29.3)	41 (70.7)	0.143
Medium	16 (40.0)	24 (60.0)	
High	52 (44.8)	64 (55.2)	

HCF = healthcare facilities; NTP = National TB Programme.

## DISCUSSION

Of the 304 HFs with documented TB cases at facility registers, 84.9% had reported cases in the LGA TB register. This is higher than the national (Nigeria) figure of 78% of HFs that reported at least a TB case to the NTP.^9^ Compared with the 2019 NTP report, the proportion of male TB patients, childhood TB rate, HIV positivity rate and the contribution of private HFs to TB cases notification are similar.^9^ The contribution of private healthcare providers to TB case-finding in Lagos increased from 12.4% in 2015 to 25% in 2019, above the national average of 14%.^9^

TB underreporting was observed in all LGAs and all types and levels of HFs. The mean percentage of TB underreporting between HF TB reports and LGA TB registers was 7.4% (14.1% to 0.9%). This is lower than earlier reports of TB underreporting of 15% between HFs and LGA TB registers in six southern states in Nigeria.^20^ The finding of 7.4% TB underreporting in this study is less than the results of other studies with TB underreporting in Cape Verde (15%), in Spanish hospitals (14.4%), in South Africa (12–20%), and in a recent inventory study in China (19.3%).^4^,^21^–^24^

The percentage difference between the aggregate case counts from LG TB registers and the State TB report was significantly lower (a mean of 1% over-reporting) than the difference between the TB facility and LG TB registers. This was due to a mix of both over-and under-reporting, with only 4/20 LG having equal reports in the state. This is unexpected, considering the quarterly data validation meetings between the state and all LG TB supervisors. A possible explanation is that LG TB registers and quarterly case-finding forms were not updated after the validation meetings.

TB underreporting was observed among all tertiary institutions and NTP non-engaged HFs. Furthermore, it was higher among private HFs than among public HFs (69.5% vs. 63.4%). Previous studies have reported similar high TB underreporting among private HFs, including complex and multiple paper-based TB reporting tools, poor clarity of reporting processes, concerns about patient confidentiality, inadequate training and feedback, and non-remuneration.^1^,^25^–^27^ It is clear, therefore, that the NTP needs to engage health providers in the design of TB reporting and recording tools, and capacity building, and consider electronic reporting using appropriate digital solutions. This finding of TB underreporting by tertiary HFs is similar to the findings of other studies due to multiple service delivery points and specialized clinics and the lack of designated facility-based TB surveillance officers to ensure effective coordination of the reporting with the designated TB clinic.^28^,^29^ A report identified non-NTP engagement and weak linkages with the non-TB part of HFs among the 10 factors for TB underreporting in analysis and quantification of TB case-finding gaps.^30^

Contrary to a study from Kenya,^28^ TB underreporting is lower in HFs and LGAs with a higher volume of patients and a high number of DOTS centers in this study. This finding aligns with an investigation in Korea by Hong et al., which showed that TB underreporting was common among the most minor HFs and low burden towns or cities.^31^ This may be because TB reporting reflected the performances of LG TB supervisors responsible for distributing all TB reporting tools to facilities. Their responsibilities include visiting all engaged HFs for supervision, data collection, updating of the LG TB register, and subsequent collation and reporting of TB data quarterly to the State program.^11^,^32^ It is possible, therefore, that the LG TB supervisors in Lagos, Nigeria, prioritized high-volume facilities and high-burden LGAs for TB reporting practices and supervision.

The TB reporting system, tools, and processes in Lagos are part of the national NTP structure, and these findings may contribute to the overall missing TB patients in Nigeria. From a health system perspective, three interventions are needed: the NTP needs to review the overall TB reporting architecture that is dependent on a physical visit by the LG TB supervisor to gather TB data at HFs, design an enabling system for ease of reporting by non-engaged HFs providing TB services, develop an internal coordination mechanism within tertiary health institutions because of multiple service delivery points, and finally, identify best practices and potential areas of linkage with the District Health Information System 2 (DHIS2) and Disease Notification and Surveillance system for notifiable diseases.

The study limitations are inherent to secondary data analysis with missing variables to link all the data sources. Data analysis and practice were retrospectives, and changes may have occurred over time in the reporting practices. Lagos has a significantly higher number of private HFs than other states; thus, our findings cannot be generalized to the entire country. A further qualitative study on healthcare workers’ perception of TB reporting and a multi-State assessment of reporting practice can support the identification of states or regional variations for targeted interventions.

## CONCLUSIONS

TB underreporting was observed between all TB reporting levels in all LGAs, and among all types and levels of HFs in Lagos. There is a need for the NTP to revise the entire TB reporting process architecture that is entirely hinged on the LG TB supervisor’s visit to all TB HFs. There is also a need to strengthen TB reporting across the board and ensure an adequate data quality assurance system through quarterly data validation meetings between HF records staff and LG TB supervisors, who should also ensure that all LG TB quarterly case-finding registers are updated after data validation meetings and link the laboratory registers to TB facility registers to assess and mitigate the initial loss to follow-up.
